# Supplementary material for: Modeling glioblastoma heterogeneity as a dynamic network of cell states
Source: Mol Syst Biol. 2021 Sep 16;17(9):e10105. doi: 10.15252/msb.202010105 (PMC8444284; doi:10.15252/msb.202010105)
Supplement: Supplementary file 6 — Source Data for Figure 5 [file MSB-17-e10105-s004.zip › Figure5A_sourcedata/GSEA_3017/nosigdb_stateF.GseaPreranked.1622795015644/TISIG_PMID_32649888.html]

Details for gene set TISIG\_PMID\_32649888[GSEA]

|  || Dataset | state33017 |
| Phenotype | NoPhenotypeAvailable |
| Upregulated in class | na\_pos |
| GeneSet | TISIG\_PMID\_32649888 |
| Enrichment Score (ES) | 0.6087859 |
| Normalized Enrichment Score (NES) | 2.9286516 |
| Nominal p-value | 0.0 |
| FDR q-value | 0.0 |
| FWER p-Value | 0.0 |
Table: GSEA Results Summary

  

Fig 1: Enrichment plot: TISIG\_PMID\_32649888      
 Profile of the Running ES Score & Positions of GeneSet Members on the Rank Ordered List

  

| PROBE | GENE SYMBOL | GENE\_TITLE | RANK IN GENE LIST | RANK METRIC SCORE | RUNNING ES | CORE ENRICHMENT || 1 | ATP1B2 |  |  | 9 | 1.759 | 0.0723 | Yes |
| 2 | HOPX |  |  | 14 | 1.345 | 0.1289 | Yes |
| 3 | SLC1A3 |  |  | 26 | 1.058 | 0.1699 | Yes |
| 4 | CSPG5 |  |  | 30 | 1.044 | 0.2139 | Yes |
| 5 | LRRC17 |  |  | 31 | 1.044 | 0.2592 | Yes |
| 6 | EDNRB |  |  | 44 | 0.957 | 0.2954 | Yes |
| 7 | MLC1 |  |  | 58 | 0.896 | 0.3284 | Yes |
| 8 | SLC1A2 |  |  | 63 | 0.857 | 0.3638 | Yes |
| 9 | AGT |  |  | 72 | 0.820 | 0.3958 | Yes |
| 10 | AQP4 |  |  | 84 | 0.789 | 0.4251 | Yes |
| 11 | RYR3 |  |  | 94 | 0.760 | 0.4540 | Yes |
| 12 | NDRG4 |  |  | 104 | 0.747 | 0.4824 | Yes |
| 13 | SCG3 |  |  | 130 | 0.698 | 0.5014 | Yes |
| 14 | NCAN |  |  | 138 | 0.686 | 0.5281 | Yes |
| 15 | CDH4 |  |  | 144 | 0.682 | 0.5554 | Yes |
| 16 | C1orf61 |  |  | 202 | 0.587 | 0.5552 | Yes |
| 17 | KCNJ16 |  |  | 227 | 0.569 | 0.5691 | Yes |
| 18 | BBOX1 |  |  | 241 | 0.559 | 0.5875 | Yes |
| 19 | ACSBG1 |  |  | 345 | 0.490 | 0.5623 | Yes |
| 20 | DPP6 |  |  | 421 | 0.462 | 0.5486 | Yes |
| 21 | SPRY4 |  |  | 443 | 0.453 | 0.5588 | Yes |
| 22 | TTYH1 |  |  | 457 | 0.448 | 0.5723 | Yes |
| 23 | FGFR3 |  |  | 515 | 0.429 | 0.5653 | Yes |
| 24 | ELOVL2 |  |  | 527 | 0.424 | 0.5787 | Yes |
| 25 | RASSF2 |  |  | 544 | 0.417 | 0.5896 | Yes |
| 26 | KCNN2 |  |  | 556 | 0.412 | 0.6025 | Yes |
| 27 | SLC6A1 |  |  | 582 | 0.404 | 0.6088 | Yes |
| 28 | FABP7 |  |  | 701 | 0.371 | 0.5717 | No |
| 29 | FAM107A |  |  | 718 | 0.367 | 0.5804 | No |
| 30 | ALDH1L1 |  |  | 744 | 0.361 | 0.5848 | No |
| 31 | LRP4 |  |  | 773 | 0.353 | 0.5875 | No |
| 32 | SDC3 |  |  | 863 | 0.333 | 0.5618 | No |
| 33 | NELL2 |  |  | 1159 | 0.284 | 0.4410 | No |
| 34 | CKB |  |  | 1200 | 0.279 | 0.4351 | No |
| 35 | PCDH8 |  |  | 1360 | 0.261 | 0.3747 | No |
| 36 | WSCD1 |  |  | 1401 | 0.256 | 0.3678 | No |
| 37 | NES |  |  | 1811 | -0.370 | 0.1994 | No |
Table: GSEA details [plain text format]

  

Fig 2: TISIG\_PMID\_32649888: Random ES distribution      
 Gene set null distribution of ES for **TISIG\_PMID\_32649888**
